# Listening to the consumer voice: developing multilingual cancer information resources for people affected by liver cancer

**DOI:** 10.1111/hex.12449

**Published:** 2016-02-29

**Authors:** Monica C. Robotin, Mamta Porwal, Max Hopwood, Debbie Nguyen, Minglo Sze, Carla Treloar, Jacob George

**Affiliations:** ^1^ Cancer Council New South WalesWoolloomoolooNSWAustralia; ^2^ Faculty of MedicineUniversity of SydneySydneyNSWAustralia; ^3^ Centre for Social Research in HealthUniversity of New South WalesSydneyNSWAustralia; ^4^ School of PsychologyUniversity of SydneySydneyNSWAustralia; ^5^ Storr Liver CentreWestmead Millennium Institute and Westmead HospitalSydneyNSWAustralia

**Keywords:** focus group discussions, health literacy, informed decision making, liver cancer, migrants

## Abstract

**Background:**

In Australia, liver cancer incidence is rising, particularly among people born in hepatitis B‐endemic countries. We sought to build an understanding of the information needs of people affected by liver cancer, to inform the design of in‐language consumer information resources.

**Methods:**

We searched the World Wide Web for available in‐language consumer information and conducted a literature search on consumers’ information needs and their preferred means of accessing it. Qualitative data collection involved bilingual researchers conducting focus group discussions (26 participants) and in‐depth interviews (22 participants) with people affected by liver cancer in English, Vietnamese, Cantonese and Mandarin. Sessions were audio‐recorded, transcribed, translated and thematically analysed. The key themes and salient findings informed the development of in‐language multimedia information resources.

**Results:**

Many consumer resources did not cater for people with low literacy levels. The participants wanted more information on cancer diagnostic and treatment options, nutrition and Chinese Medicine and experienced communication challenges speaking to health professionals. While Vietnamese speakers relied entirely on information provided by their doctors, other participants actively searched for additional treatment information and commonly used the Internet to source it. We developed multilingual, multimedia consumer information resources addressing identified consumer information needs through an iterative process, in collaboration with our multilingual consumer panel. These resources are available in four languages, as separate modules accessible online and in DVD format.

**Conclusion:**

This process enabled the development of user‐friendly patient resources, which complement health‐care provider information and supports informed patient decision making.

## Background

Until recently, liver cancer, also known as hepatocellular cancer (or HCC), was a relatively rare cancer in Australia, but its incidence has been steadily rising over the last decades.^1^, ^2^ As its 5‐year survival rate remains one of the lowest for any cancer (approximately 15%),^3^ liver cancer is now one of the top 10 local causes of cancer death.^4^ HCC disproportionately affects people born in hepatitis B‐endemic countries, with 47% of all diagnoses in the largest Australian state (New South Wales) affecting residents born overseas.^5^ People born in China, Macau, Hong Kong and Vietnam are 6‐ to 13‐times more likely to develop HCC than other (non‐indigenous) Australians.^5^ As many live in Sydney's south‐west, this region has the largest burden of liver cancer in Australia.^6^

A case series from the local teaching hospital found that 41% of their patients diagnosed with HCC were Asian‐born, 75% of them presented late, and overall median survival was just 5.1 months.^7^ This region has the poorest SEIFA (Socio‐Economic Index of Disadvantage for Area) indicator in Sydney.^8^ As 30% of the local population speaks another language and little or no English,^8^ low language and health literacy levels^9^ limit migrants' ability to engage with the health system^10^, ^11^ and could contribute to poor HCC outcomes.^7^

Data from California suggest that people of Asian ancestry with HCC presented at a more advanced disease and were less likely to receive treatment.^12^ Vietnamese residents’ limited English proficiency, lower socio‐economic status and educational levels^2^ were significant barriers to navigating the health‐care system.^13^

As besides English, Vietnamese, Mandarin and Cantonese are the dominant languages spoken by Australians affected by chronic hepatitis B (and HCC),^10^ diagnostic and treatment information should also be available in these languages. Given the short survival experienced by the vast majority of patients diagnosed with HCC, this information should be readily accessible at the time of diagnosis. A research prioritization exercise carried out by a local cancer charity (Cancer Council New South Wales) identified a general lack of information resources and support systems for people affected by liver cancer. It recommended the development of culturally appropriate HCC information resources in English and languages spoken by people commonly affected by HCC. The consultation also noted the paucity of local research into Asian refugees and immigrants’ understanding of viral hepatitis and liver cancer.

Consequently, this study sought to build a better understanding of the unmet information needs experienced by people affected by liver cancer. The findings informed the process of development of tailored consumer information resources in English, Vietnamese, Cantonese and Mandarin.

## Methods

The study was approved by the Human Research Ethics Committees of the south‐west Sydney and Western Sydney Area Health Services and was conducted in four stages. They included: (i) identifying existing consumer information resources on liver cancer in English, Vietnamese and Chinese languages, (ii) a literature review on the information needs of people affected by HCC and preferred ways of accessing them, (iii) qualitative research with people affected by liver cancer and (iv) developing multilingual, multimedia user‐friendly resources about liver cancer.

Information collected at each step informed the development of subsequent stages.

### Identifying available consumer resources on liver cancer

Bi‐lingual Cancer Council staff and volunteers conducted Internet‐based searches to identify fact sheets, booklets, pamphlets, brochures and Web pages providing consumer information about liver cancer diagnosis and treatment in English, Chinese languages and Vietnamese. The information was collated and reviewed utilizing the quality assurance process used for developing Cancer Council New South Wales’ (CCNSW) Cancer Directory Website: http://www.cancerdirectory.com.au/home/process.

### Conducting the literature review

The literature search sought to: (i) explore the viral hepatitis‐ and cancer‐related knowledge and beliefs of Vietnamese and Chinese people, (ii) identify strategies to address the health information needs of Chinese and Vietnamese immigrants and (iii) ascertain consumers’ preferred sources of health information. A search of the academic literature was conducted using *Sirius*, a search engine linked to social science, public health and medical databases including PsycInfo, Medline, Science Direct, Web of Science and Social Sciences Citation Index. We conducted a separate search of the PubMed Central database. To uncover relevant research articles, we carried out keyword searches using the following terms: viral hepatitis, hepatitis B, hepatitis C, hepatocellular carcinoma, primary liver cancer, treatment, information, support, health promotion and Australia. We also reviewed the grey literature and health promotion resources.

### Consumer consultation with people affected by liver cancer

#### Participants and recruitment procedures

The consultation sought to involve people affected by liver cancer, defined as individuals diagnosed with HCC and those caring for them. Prospective participants were identified among people with HCC attending liver clinics at two major teaching hospitals in Sydney, or the private consulting rooms of a Vietnamese‐speaking liver specialist. Recruitment of Chinese participants used the networks of CanRevive Inc., a cancer support organization providing services to the Chinese community in Sydney. Bilingual researchers contacted potential participants who expressed interest in the consultation process, provided additional information, answered questions about the study, obtained participant consent and ascertained their preferences regarding ways of becoming involved in the consultation.

#### Procedure

The community consultation was guided by community‐based participatory research principles^14^ and engaged consumers at all stages of the research and resource development process. Focus group discussions (FGDs) were based on standard methodology, and a focus group guide was developed by investigators in collaboration with clinicians and consumers.^15^ While using FGDs offers more flexibility and facilitates the exploration of a broader range of attitudes and feelings, our group resolved that in recruiting participants with a terminal illness in a consultation process, prospective participants should be given a range of options to match their condition, so participants could choose to participate in either FGD, face‐to‐face interviews or telephone interviews in their preferred language. We collected de‐identified information on participants’ age, gender, length of Australian residence and highest educational attainment. Similar questions were developed for FGDs and for in‐depth interviews (IDI) (see Table 1).

**Table 1:** Sample interview/FGD questions guide used in the community consultation

What information did you receive about your liver cancer diagnosis and treatment and how was it delivered? Did you feel you needed more information? Where did you look for it and how easy was this to find? On what topics would you have liked more information? Foods to eat/avoid, what exercise to take and changes to your routines Practical support (accessing health benefits, household assistance, etc.) Symptoms needing medical attention. Did you receive some take home information and in what format? Was information provided in your own language and was it useful? Where else did you look for more information? Was this easy to find? What is the best format for getting patient information about cancer? What information should be included when developing new resources for liver cancer? Were your particular information needs well met? What is your advice for people developing patient information resources?

The research team conducted all FGDs and IDI, with bilingual researchers conducting the Vietnamese, Cantonese and Mandarin qualitative data collection. FGDs took place in venues close to cancer treatment centres attended by participants and IDIs took place in various locations selected by participants (usually home, or specialist's offices) or by telephone. Topics covered in the consultation included participants’ experience of a liver cancer diagnosis, their perceptions of the quality and quantity of information received and its timeliness. We also enquired whether they had a good understanding of investigations, staging and treatment options received, what information sources they used and their preferred ways to access information.

Interviews and FGDs were recorded, translated, transcribed and verified. The approach to analysis of interview and focus group data was informed by Braun and Clarke's guidelines for conducting thematic analysis.^16^ Data were reviewed by all investigators and main themes and key issues identified and grouped by topic and by language. The investigators used these findings to derive a series of ‘actionable findings’ which informed the development of consumer resources.

### Developing multimedia resources on liver cancer

The ‘actionable findings’ identified in the previous stage included recurring themes, questions and practical issues raised by research participants. Answers were scripted, based upon information provided by clinicians and after cross‐referencing with consumer resources identified through Internet searches. These answers underwent consumer review, to ensure they avoided medical jargon. In developing resources, we used the health literacy universal precautions approach, which assumes that all patients are at risk of not understanding their health condition and require patient‐friendly and culturally appropriate support to do so.^17^ Liver specialists, nurses and consumer representatives critically reviewed the scripts and the revised versions were translated into Vietnamese, Mandarin and Cantonese. Bilingual content experts and consumer representatives reviewed the translations to ensure technical accuracy and cultural appropriateness.

Subsequently, the scripts were video‐recorded in all four languages by bilingual health‐care providers, people affected by HCC and other consumer representatives. The recordings were structured into modules, addressing the topics prioritized through consumer consultation. Finally, in‐language screen captions were added to the video clips, summarizing the key messages of each segment.

## Results

### Internet searches

Internet searches identified a wide range of information resources (mostly web pages, fact sheets and booklets) on liver cancer hosted on Australian, North American and UK websites. They largely met Cancer Council's quality assurance criteria for consumer publications and provided information on liver cancer risk factors, disease symptoms and signs, information about a HCC diagnosis, staging, treatments and side‐effects. As the vast majority consisted of written materials, this relied upon users having good literacy skills. We identified several good quality HCC resources on Chinese websites, but very limited resources in Vietnamese. A resource list was compiled, including web links and a brief appraisal of the information. Overall, our searches found that few of these resources were designed for people with low literacy.

### Literature review

As the original search revealed limited Australian research about Chinese and Vietnamese migrants’ perceptions of liver cancer, we broadened this to include the international literature. Articles were included in the final review if they addressed one or more of the topics of interest listed in the ‘Methods’. As few papers answered our original research questions, we broadened the search to include migrants’ health seeking and scanning practices pertaining to any cancer, as well as to viral hepatitis.

A systematic review of papers published from 1980–2002 examined cancer patients’ information needs and their preferred sources of cancer information.^18^ The review found that at all disease stages, patients primarily sought treatment‐related information, specifically related to disease stages, treatment options and treatment side‐effects.^18^ Most commonly used information sources were printed materials (34.6%) and health professionals’ advice (26.9%).^18^ The study concluded that ‘In general, there do not appear to be dramatic differences in information needs or sources based on racial/ethnic differences’.^18^

Our own literature review (M. Hopwood, Personal communication) identified 48 peer‐reviewed research articles in the English literature published from 2002 to 2014 and suggested differences in patients’ information needs and sources based upon ethnicity. Despite Chinese–Australians being more likely to access mainstream Australian media than Vietnamese Australians, patterns of access to health‐related information within both communities were largely similar and involved ethnic media and community networks. Importantly, the literature review highlighted a need for developing culturally relevant health promotion materials that take account of ethnically patterned health beliefs when addressing chronic viral hepatitis transmission, blood borne virus testing and screening for primary liver cancer among Chinese and Vietnamese Australians.

### Consumer consultations

From April 2013 to February 2014, we conducted FGDs and IDI with 48 people affected by liver cancer (see Table 2 for a summary of participant demographic characteristics). The four FGDs involved 26 participants; two were conducted in English (one of them involved Australian‐born and one overseas‐born English speakers) and one each in Cantonese and Mandarin. Twenty‐two people took part in IDIs; as all Vietnamese participants preferred the IDI format, no FGDs took place in this language.

**Table 2:** Summary characteristics of participants in the consumer consultation process

Participant characteristic					Overall
Preferred language	Cantonese *n* = 12	Mandarin *n* = 6	Vietnamese *n* = 11	English *n* = 19	*n* = 48
Mode of interaction					
Focus group discussions	8	3	–	15	26
In‐depth interviews	4	3	11	4	22
Gender					
Male	5	4	9	14	32
Female	7	2	2	5	16
Median age (years)	75	56	61	58	64
Median duration of Australian residence (years)	24	26	23	56	25
Highest educational level	*n* (%)	*n* (%)	*n* (%)	*n* (%)	Overall (%)
Primary school	4 (33)	–	5 (50)	2 (25)	11 (31)
High school	5 (42)	1 (17)	4 (40)	–	10 (27)
University or postgraduate studies	3 (25)	5 (83)	1 (10)	6 (75)	15 (42)
Not documented	–	–	1	11	12

#### Participants’ information needs

Similar to the findings of Rutten *et al*.,^18^ people affected by liver cancer wanted to access more detailed information about their disease. Overall, our participants sought more specific information on liver cancer staging, treatment and prognosis, to help them make sense of their disease, treatment and outcomes. Understanding their condition was challenging and participants reported taking along family members to consultations to clarify what they heard and at times to act as their informal interpreters. They also relied on their GPs or hepatology nurses to explain the significance of test results and asked for clarifications from other patients.

Patients generally acknowledged that doctors were willing to answer their questions, but remained cognizant of doctors’ time constraints, which limited the time they had to do so. The use of medical jargon posed particular challenges, especially for non‐English speakers. Reliable information about treatments and side‐effects was difficult to source and patients spoke about learning more from other patients.

…[I]t would be good if there could be a monthly magazine introducing us to new medications, describing its effects and side effects, because otherwise there is no way for us to find out. (Cantonese‐speaking participant)

Irrespective of language group, participants expressed a need for more practical and psychosocial support.

Yes, how to communicate with family and also encourage [patients] to participate in social gatherings with other cancer patients. (Cantonese‐speaking participant)

Recurrent topics included information about financial matters, transport and difficulties in accessing the services of interpreters and social workers.

… [W]e don't even know that these [transport and financial support] services exist. It would be good to have transport to the hospitals. (Mandarin‐speaking participant)

#### The role of nutrition and Chinese Medicine

All participants, but in particular Chinese and Vietnamese speakers, wanted to know how Chinese Medicine and/or herbal medicine could complement the medical care they were receiving.

Participants wanted more information about diets and nutrition suitable to their condition. Many participants searched for advice on types of dietary modifications following diagnosis and their cultural norms and family's advice shaped their decisions in this respect.

I personally think that there are a lot of needs, a nutritionist included. Everyone is different in their needs. Not all the vegetables and fruits are suitable for everyone. Some people may have allergies. (Mandarin‐speaking participant)

A recurrent theme in the Vietnamese consultation was that coping with liver cancer was facilitated by focusing on keeping a ‘peaceful mind’. Future interventions that could facilitate ‘a peaceful mind’ might include information about how to practice or supporting the use of meditation, yoga and Tai chi. Information about a range of techniques that help manage stress and promote relaxation, such as listening to traditional Vietnamese music might also be recommended.

#### Communication barriers

Chinese and Vietnamese participants identified a lack of familiarity with the English language as a major barrier to negotiating local health services and for understanding the implications of a cancer diagnosis.

So the GP referred me to an oncologist. But he does not speak Chinese. I do not know English. So my daughter had to come with me every time. Each time in the consultation he would be talking to my daughter and then my daughter would explain it back to me afterwards. (Cantonese‐speaking participant)

Communication was at times difficult, even when speaking to Chinese‐speaking doctors, as GPs offered limited information about available treatments (although they were the best resource for explaining hospital discharge instructions and related information). Participants suggested that the ways in which ‘bad news’ is communicated to patients and carers should take into account patients’ limited English language proficiency and cultural norms.

The specialist diagnosed me with liver cancer… my mind was in such a chaos, I did not know what to think at all. [Other interviewees agreeing in the background] … Firstly, the conversation was in English, so there were a lot of nouns that I have never heard of in my life and that really reduced my understanding … The doctor also said it very simply and quickly … at moments like these, if there is someone who can speak your own language, then it would've been a lot better. (Cantonese‐speaking participant)

Few non‐English speakers were aware of the existence of a telephone interpreting service and/or how to access it. Chinese participants suggested compiling a directory of Chinese‐speaking medical practitioners and to make this available in hospitals, GP and specialist clinics. Chinese participants also recommended publicizing the availability of interpreter services.

There is a language barrier. My daughter doesn't know medical jargons so even when her English is good, she cannot translate properly. (Mandarin‐speaking participant)

#### Participants’ preferred sources of cancer information

Overall, research participants believed that medical practitioners were their best sources of authoritative health information, but also wanted to independently access information:

…it would be useful to have all this information condensed in one place where, you know, a website that is dedicated to liver cancer where you can go in and find whatever you want like, you know, search functions and things like that? Do you, does anybody have any ideas? (English FGD participant)

While clinic nurses, social workers and patient support groups were seen as very valuable sources of practical advice and assistance, all language groups saw the Internet as a good source of information:

The information that the doctor gave me was in English, I don't know what it is in Chinese. So I looked on the internet, there is access to information worldwide. It is quite useful because we can search up the jargon because my daughter doesn't know medical jargon either. (Cantonese FGD participant)

Vietnamese participants were largely disinclined to seek cancer‐related information outside medical encounters and were unanimous in reporting medical practitioners as their preferred source of cancer‐related information. Most believed that looking further afield for cancer‐related information was unnecessary, as the information they received from their doctors was tailored to their individual condition, unlike other sources of information, which were too generic to offer any personal benefit or meaning.

However, some Vietnamese participants also used the Internet as a means of gathering additional detail, particularly after receiving cancer treatment:

After the operation, I searched for the information myself. In this Internet Age, I went to the Internet visiting English websites and if my English is not enough I went to Vietnamese websites. (Vietnamese participant)

In general, the most useful sources of information participants could source were printed materials, DVDs and USBs provided by pharmaceutical companies. Some participants had consulted the Cancer Council's ‘Understanding liver cancer’ booklet, which they felt offered good generic information about cancer, but lacked the detail required on specific diagnostic and treatment options.

A small booklet [everyone agrees]. We can read it whenever we want. It is more convenient. A CD can be very tiring for a patient after a while. (Cantonese‐speaking participant)

Many participants used the Internet for researching information about new treatments, complementary medicines and clinical trials. In‐language cancer support groups and information services provided by a Chinese patient support organization (CanRevive Inc., Sydney). Participants also referred to the stigma associated with a cancer diagnosis and the community perceptions that cancer is contagious. They felt the support provided by this patient support organization helped them to transcend the feelings of social isolation they experienced.

### Resource development

The process of resource development was iterative and time‐intensive, relying on multiple rounds of feedback and revisions informed by expert and consumer reviewers. Specific issues raised by participants were tabulated and grouped under ‘actionable findings’ and frequently asked questions (FAQ) by topic and by language, to ensure they were addressed during the resource development phase.

As many participants stated they were using the Internet for information about cancer treatments, we opted for an online video format to address questions arising from the consumer consultation. The information in these resources is being delivered by bilingual health professionals (doctors and nurses), in line with preferences expressed by our reference group. Using medical experts as speakers was a means to ensure the information presented was legitimate and credible. As participants wanted information resources that can keep pace with the rapidly evolving field of liver cancer treatment, we opted for an online format, which is easier to keep current than printed materials, which are also more expensive to produce.

The video segments were recorded in four languages and grouped into 10 modules (for a topic listing, see Table 3), which addressed all the key points raised in the consumer consultation. They are available online, on You Tube and on the Cancer Council website, as well as in a DVD format.

**Table 3:** List of the liver cancer multimedia resource modules developed

Module #	Topic
1	About liver cancer
2	Screening for liver cancer
3	Diagnosing liver cancer
4	Non‐surgical treatments for liver cancer
5	The surgical treatment of liver cancer
6	Treating liver cancer: disease and treatment complications
7	Complementary and alternative medicines and nutritional issues in liver cancer
8	Hepatitis and liver cancer
9	The liver in health and disease – more on hepatitis B
10	Support for people affected by cancer

To ensure these resources could reach their intended audience, DVDs and advertising leaflets and posters containing the website links to resources are distributed through liver clinics in public hospitals and through the networks of the relevant medical and nursing associations.

## Discussion

This study describes a process of developing multilingual information resources for people affected by liver cancer, informed by community consultation. We sought to meet the cancer information needs of people with varying levels of health literacy and reflecting the linguistic demography of people affected by liver cancer in Australia. As the average educational attainment and English proficiency levels were relatively low, patients and their families experienced additional challenges in understanding the disease and navigating the health‐care system.^13^

As all participant groups wanted more information about liver cancer diagnostic and treatment modalities, we developed stand‐alone modules addressing these topics. Other priority topics discussed the role of diet and Complementary Therapies, especially Chinese Medicine, in cancer treatment and included information on ways to access practical support. We also included sections on the link between hepatitis and liver cancer, to inform patients’ close contacts how to protect themselves against viral hepatitis.

The resource development process was underpinned by the tension obvious in the literature review and community consultation: treating doctors are viewed as the primary source of information about HCC, although people with low English language skills experience difficulties communicating with their doctors and are concerned about the limited time that they have during the consultation. Without English‐speaking skills (and with low literacy levels), it is challenging to access cancer‐related information through the mainstream Western media, or via educational pamphlets and interaction with English‐speaking health‐care professionals. Similar to findings of previous research, our Vietnamese‐ and Chinese‐speaking participants experienced problems communicating with their treating doctors about cancer, as well as experiencing language and translation difficulties.^19^, ^20^, ^21^ A recent Australian article described the information needs of culturally diverse cancer survivors. Respondents commented on insufficient information about available practical support, how to navigate the health system, difficulties in communicating with health professionals, misunderstandings about disease management and the need for more cancer‐related information on prognosis and treatment.^22^

As doctors’ advice was highly valued by all language groups, we developed visual resources that can complement the information provided in a medical encounter, without relying on advanced literacy skills.

According to an Australian study, patterns of accessing health‐related information were different among Chinese and Vietnamese immigrant populations living in Brisbane.^23^ As culturally and linguistically appropriate information materials written in Vietnamese were difficult to source, this could have reinforced Vietnamese people's preference to seek all their health‐related information from their family doctor, whom they trusted beyond all other sources. O'Connor *et al*.^24^ corroborated our finding that the Vietnamese community in Australia relies heavily on Vietnamese family doctors for information about health‐related matters. However, given the short duration of the average medical consultation, Vu *et al*.^23^ concluded that Vietnamese family doctors were not always ideal sources of information. As Vietnamese participants were satisfied to receive all information from their Vietnamese‐speaking doctors, providing patient information resources through Vietnamese‐speaking doctors can be a valuable dissemination strategy for this group.

To some extent, Vietnamese participants’ reluctance to search more widely for information may be attributable to their educational levels: half had attended only primary school education and the remainder high school. Of note, the practice of asking patients their educational attainment (as a proxy for health literacy levels) can underestimates the literacy levels in a population,^25^ as education levels often underestimate patients’ reading levels.

Several studies of health information seeking practices examined the value of the Internet as a model for promoting health literacy around conditions such as cancer and hepatitis. A US study of Chinese American cancer survivors with limited English skills concluded that the Internet is probably not widely accessed by non‐English speakers; they suggested that health information should be provided in a variety of media, in patients’ maternal language.^26^ However, an Australian study examining Internet usage among primary care patients found that online health information seeking is prevalent among Australian general practice patients. Patterns of use varied with age and socio‐economic status, but not by patient gender, English‐speaking background status and geographic location. The proportion of Internet users and online health information seekers decreased with age, yet more than half (55%) of all patients aged 65–74 years used the Internet, about one in five (21%) sought health information online, and one in 10 (10.5%) obtained information online about a problem prior to seeing the GP about it. While the most advantaged patients were twice as likely to use the Internet, online health information seeking was not uncommon even among the most disadvantaged patients.^27^

We developed information resources in a visual format and invited consumer feedback at all stages of the project. This aligns with the World Health Organization recommendations on strategies to assist the health literacy needs of migrants, which suggests that migrant users should be involved in the planning, implementing and evaluating processes and emphasizing the importance of employing plain language and visual imagery.^9^

Beyond language proficiency, other barriers to seeking and using health‐related information involve culturally based values and beliefs, including a preference to use folk remedies or Chinese Medicine,^28^ particularly if Western medicine was perceived as ineffective.^28^, ^29^ As all language groups expressed interest in Complementary and Alternative Medicine, we included a module on its role (and caveats) in cancer care.

The lack of familiarity with Western health‐care systems and processes identified by other researchers^30^, ^31^ was a recurrent theme in our consultation. Consequently, the multimedia resources attempted to fill these gaps by including in‐language information about the Western health systems and what to commonly expect when attending a hospital for cancer treatment.

Our literature review indicated that awareness of language and culture must be salient in the development of health promotion materials in Asian‐immigrant populations. Similar to Yiu and Kirsner,^32^ we found that Chinese participants mentioned the stigma associated with a cancer diagnosis and the community perceptions that cancer is contagious. They thought that some of their friends avoided them, exacerbating their feelings of social isolation, a fact noted also by others.^33^

Study limitations include the relatively small participant sample across the four language groups targeted and the attending risk of sampling bias. While the themes identified through focus groups and IDI were broadly similar for all language groups, we cannot rule out that the differences in methodology could have influenced the type and depth of data produced through each approach. The different strategies used to recruit Vietnamese‐ and Chinese‐Australian participants may have implications for the generalizability of study findings to all people from these populations with liver cancer. However, qualitative methods are not used to make generalizable claims, but rather to better understand participants’ experiences. It could be argued that the purposive sampling technique used (recruiting participants from support services organizations, as well as from hospital clinics, and the use of both focus groups and IDI enhanced the amount of information generated by the participants. Focus groups discussions stimulated participants to talk about their own experiences and encouraged the sharing of ideas and perceptions. The IDI generated individual views, free of peer pressure and need to conform and were less intimidating for less articulate or introverted members we contend that a combination of these methods produced a richer data set. We acknowledge that in exploring patients’ perception regarding their unmet needs, interviewers asked specific clarifying questions, if this did not arise spontaneously during the discussion, given that CALD patients are less likely to openly express their unmet needs. We acknowledge this as a study limitation.

The commonalities of themes and issues raised by participants across languages and methods of enquiry are worth of note, however, particularly as they also echoed the findings of the literature review.

## Conclusion

Our work has mapped out a stepwise process of developing multimedia consumer resources informed by consumer consultation. They address the information needs of people with limited health literacy and were produced in languages reflecting the linguistic demography of hepatitis B^10^ and liver cancer^34^ in Australia. The treatment information allows viewers to consolidate their understanding of their diagnosis, filling in gaps that may arise during often‐brief clinical encounters. The information modules offer consumer‐friendly information recorded in‐language by bilingual clinicians, representing what Chen *et al*.^28^ referred to as ‘high royalty’, that is dependable cancer information. Participants in our consultation highly valued the ability to access information and supportive care in their own language and where available, to access the social networks offered by ethnic patient support organizations. Therefore, the provision of such services should be an integral part of the delivery of cancer information and support services in migrant communities.

## Conflicts of interest

None declared.

## Funding support

This work was funded by Cancer Council NSW and by Cancer Australia through the *Supporting people with cancer* Grant initiative, funded by the Australian Government (Project ID CA‐A1112/49). We also received support from the Sydney West Translational Cancer Research Centre (TCRC), funded by the Cancer Institute NSW. Jacob George is supported by the Cancer Council NSW, the Robert W. Storr bequest to the Sydney Medical Foundation and grants from the National Health and Medical Research Council (NHMRC). The Centre for Social Research in Health is supported by a grant from the Australian Government, Department of Health.
